# Advanced Analysis Tools for Two Wavelength Autofluorescence Imaging of Macular Xanthophyll Carotenoids: ALSTAR2 Baseline

**DOI:** 10.1167/tvst.14.8.32

**Published:** 2025-08-21

**Authors:** Andreas Berlin, Lukas Goerdt, Mark E. Clark, Liyan Gao, Thomas A. Swain, Gerald McGwin, Cynthia Owsley, Kenneth R. Sloan, Christine A. Curcio

**Affiliations:** 1 Ophthalmology and Visual Sciences, The University of Alabama at Birmingham, Heersink School of Medicine, Birmingham, AL, USA; 2 University Hospital Wurzburg, Wurzburg, Bavaria, Germany; 3 Department of Ophthalmology, University Hospital Bonn, Bonn, Germany; 4 Department of Epidemiology, School of Public Health, University of Alabama at Birmingham, Birmingham, AL, USA

**Keywords:** aging, age-related macular degeneration, autofluorescence, xanthophyll carotenoids, fovea, automated image analysis software, centration, HILLCLIMB, contour analysis, ImageJ, optical coherence tomography, Müller glia, retinal pigment epithelium

## Abstract

**Purpose:**

To allow exploration of xanthophyll carotenoids in vision and age-related macular degeneration progression using two-wavelength autofluorescence imaging for macular pigment optical density (MPOD), we developed tools for automatically centering and classifying the MPOD distribution pattern.

**Methods:**

A subset of the ALSTAR2 baseline cohort (NCT04112667) and 44 eyes of adults aged 20 to 30 years with healthy maculas were imaged with optical coherence tomography and two-wavelength autofluorescence (MPOD module, Heidelberg Engineering). Images underwent a quality review. Two custom FIJI plugins centered the MPOD distribution by five algorithms (FOVEA, HILLCLIMB, CENTROID, MAX, CONTOUR). Others automatically classified spatial distributions into four patterns from Obana et al: Peak, Ring, Mixed, and Dip.

**Results:**

Of 651 qualifying aged eyes and 44 young eyes, the HILLCLIMB and CONTOUR methods best agreed with a manually determined foveal center. Regarding spatial distribution pattern, 445 aged eyes (68.4%) showed peaks, 118 (18.1%) rings, 41 (6.3%) mixed, and 47 (7.2%) dips. In young eyes, 40 (90%) showed peaks, 1 (2.3%) rings, 3 (6.8%) mixed, and none showed dips. Notably, peaks were significantly (*P* < 0.001) more prominent in men (74.1%) than women (65.0%) and pseudophakic (72.7%) than phakic (62.9%) eyes.

**Conclusions:**

Automatic tools for MPOD centration are reliable and robust. Future studies will use the HILLCLIMB and CONTOUR algorithms.

**Translational Relevance:**

Automated MPOD pattern assignment suggests that the spatial distribution of MPOD varies with gender, lens status, and possibly age. Our analytic software can be applied to large samples for studies of xanthophyll carotenoid impact on vision and age-related macular degeneration progression.

## Introduction

Macular xanthophyll carotenoids (MXCs), primarily lutein and zeaxanthin,^1^^,^^2^ are dietary micronutrients that protect brain and retinal cells by absorbing blue light, decreasing oxidative damage, and performing other functions.^3^^,^^4^ Exclusively supplied by diet, lutein and zeaxanthin are selectively transported to the retina via high-density lipoprotein complexes.^5^^,^^6^ With an ocular tissue metabolite meso-zeaxanthin, they form the macular pigment (MP)^7^ in the fovea and surrounding macula lutea.^8^ Directly visualized MP localizes anterior to the external limiting membrane (ELM),^9^ with a precise spatial distribution supported by carotenoid-binding proteins.^10^ Several methods exist^11^^–^^15^ to measure the MP optical density (MPOD). These enable the monitoring of MP concentration^16^ and distribution^17^^–^^20^ in response to dietary interventions^21^^,^^22^ or neurodegenerative conditions like macular telangiectasia^23^^–^^25^ and age-related macular degeneration (AMD).^22^^,^^26^^–^^28^

Autofluorescence (AF) is a valuable tool to visualize MP in the central retina. AF captures strong emissions elicited from the retinal pigment epithelium organelles after excitation by blue light (488 nm wavelength). MP absorbs incoming blue light, which lowers detection of emissions from the retinal pigment epithelium. Hence, retinal areas where the MP is present appear dark on AF. To circumvent the impact of MP on AF, green AF (514 nm) has been introduced, which is outside the absorption spectrum of MP. Different approaches to MP quantification using either single-wavelength^12^^,^^29^ or two-wavelength AF (2WAF) have been explored.^13^^,^^17^^,^^30^ Regarding 2WAF, MPOD is defined as the log_10_ ratio of green emissions to blue emissions at each pixel, compared with a reference point where the MPOD is near zero (9° from the foveal center).^15^^,^^31^ Compared with psychophysical tests like color matching, AF-based approaches are fast, objective, and reliable, and all pixels are available for analysis rather than selected test locations.^32^ These techniques allow MPOD distribution patterns to be identified, quantified, and compared with underlying anatomical correlates. Herein, we use MXC as an abbreviation for the chemical class, 2WAF as an imaging technology for MP, and MPOD as a metric for 2WAF.

**Figure 1.**
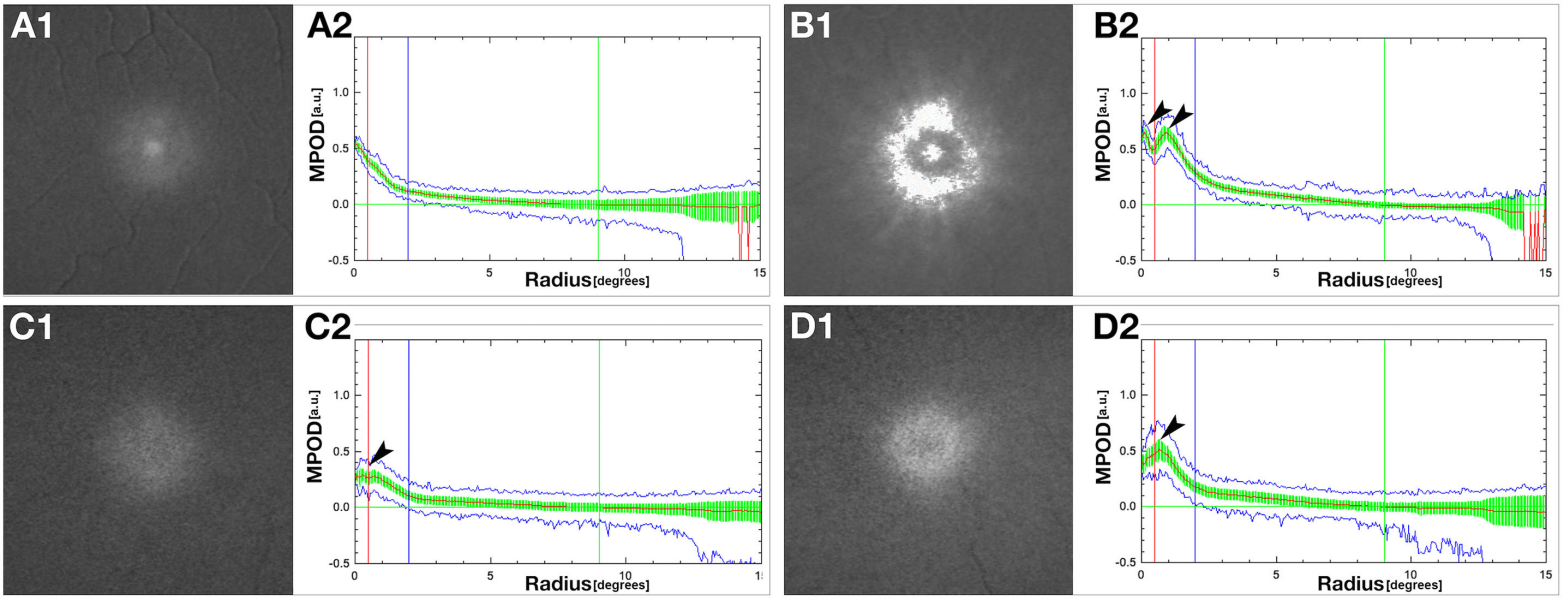
Representative patterns of MP distribution. Study eyes (see Methods) are classified in patterns described by Obana et al.,^20^ expressed as averaged across all meridians (format of the manufacturer, Heidelberg Engineering, Heidelberg, Germany), and described using a simplified terminology. At the left of (**A**–**D**) is a 2WAF image. To the right of (**A**–**D**) is the mean MPOD averaged over all meridians. Standard deviations (*green bars*) and maximum and minimum values (*blue lines*) are shown for each eccentricity. Radius is the distance from the foveal center (inward rise of the ELM) in degrees. The *red line* indicates a 0.5° radius (1° diameter circle, ∼0.288 mm). The *blue line* indicates a 2° radius (within the parafovea or inner ring Early Treatment Diabetic Retinopathy Study). The green line indicates the 9° reference distance for calculating MPOD (see Methods). (**A1**–**2**). Peak: MPOD density profile peaks at the foveola and declines a monotonically with increasing distance from the foveola (eccentricity). (**B1**–**2**) Ring: The MPOD density profile has two maxima, one at the foveal center, and another at 0.8°–1.0° eccentricity that represents the crest of a parafoveal ring (black arrowheads in **B2**). (**C2**) Mixed: The MPOD density profile shows a plateau at the fovea (black arrowhead in **C2**). (**D2**) Dip: The MPOD density profile is lower in the foveal center compared with a higher density ring in the surrounding area (black arrowhead in **D2**).

Investigators using single-wave AF^29^ and 2WAF^17^^,^^18^^,^^20^^,^^33^ have described the MPOD distribution as having annuli of alternating high and low AF signals centered on the fovea. Obana et al.^20^ termed these patterns central peak, ring-like, intermediate, and central dip (Fig. 1). Central peak has a single peak at the foveola, with a monotonic decline toward the periphery (Fig. 1A). Ring-like has two distinct peaks, one at the foveola and another along the crest of the surrounding parafovea (Fig. 1B). Intermediate has a central plateau (Fig. 1C). Finally, central dip has a reduced density at the foveola, with a higher density in the surrounding parafovea (Fig. 1D). Although Obana et al.^20^ analyzed the relationship between these patterns, foveal shape, and layer thickness on macular optical coherence tomography (OCT), MPOD images were not directly linked to an anatomical foveal center on OCT. Although indwelling software automatically determined a center,^34^ occasional misalignment, particularly in central dip types, required manual adjustment.^20^

**Figure 2.**
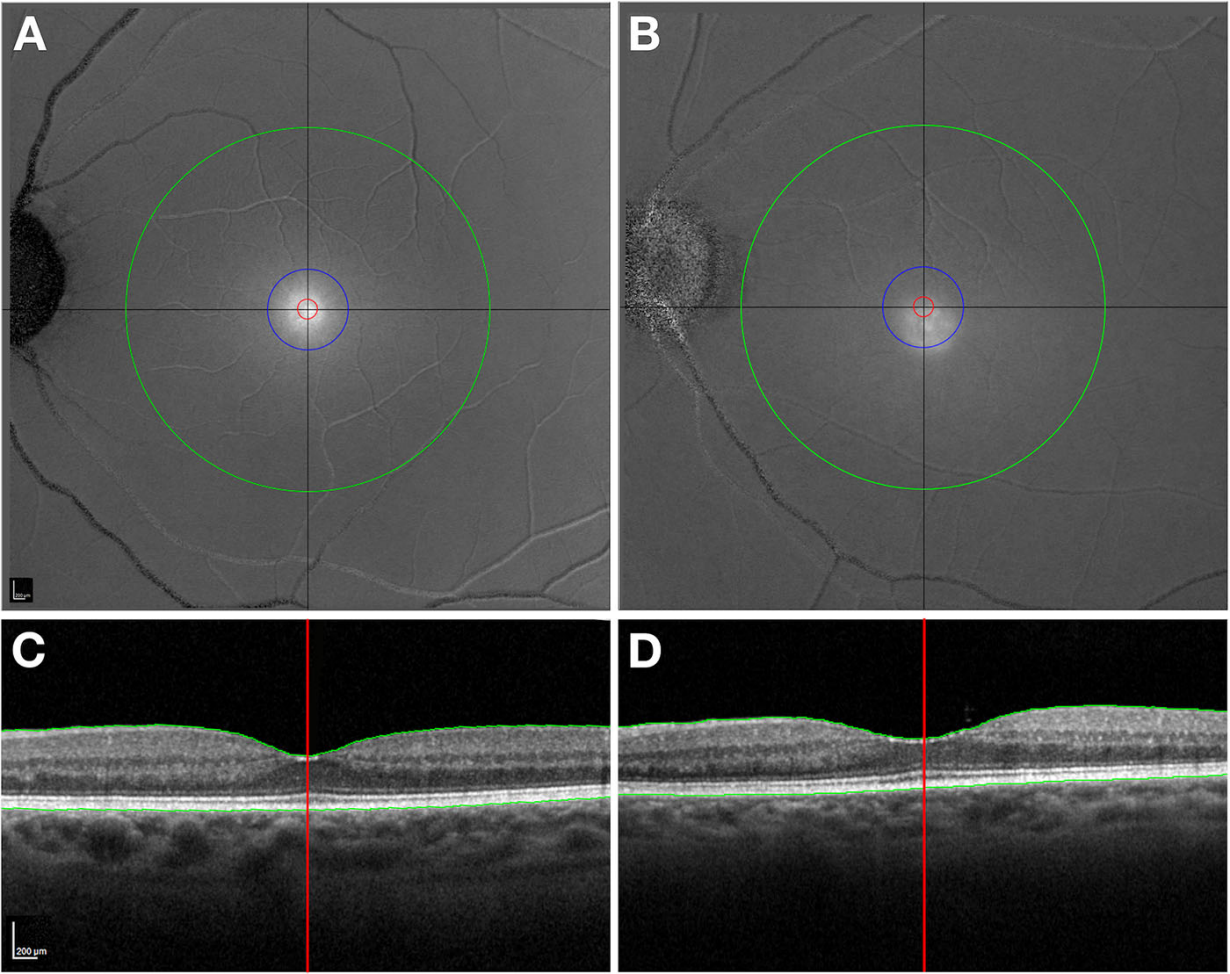
The anatomical foveal center and the center of the MPOD distribution may be discrepant. (**A**, **B**) MP distribution in gray scale. (**C**, **D**) An anatomical foveal center is visible in OCT B-scans. The inward rise of the ELM, or external fovea of classic neuroanatomy,^51^ is the location of the longest cone photoreceptors in the central bouquet. **(A**, **C**) The center of the MP distribution corresponds with the external fovea. **(B**, **D**) The center of the MP distribution does not align with the external fovea. The MP distribution is shifted temporally and inferiorly.

Accurate determination of the MPOD center is crucial, because it may not align with an anatomical foveal center seen in OCT (Fig. 2).^35^^,^^1^^,^^36^ Misalignment is likely due to developmental cellular rearrangements that create the foveal pit and high cone density in the human retina on independent time scales*.*^37^^,^^38^ Precise centration can ensure consistency in MPOD analysis and facilitate the harmonization of metabolic changes with structural changes observed using OCT. Automated centration is essential for standardizing measurements, minimizing interobserver variability, and providing a reliable reference point for subsequent analysis. Altogether, precision in imaging can assist the exploration of hypotheses linking xanthophyll bioavailability, the high-density lipoprotein pathway of AMD genome-wide association studies, and foveal shape as specified by development.^39^ ^27^^,^^40^^–^^42^ This information can in turn inform new intervention trials and public health dietary recommendations.

To comprehensively investigate the role of MP in aging and AMD, we developed tools for centering and classifying MPOD distribution patterns based on the Obana et al.^20^ description. Results obtained in a large cohort of aged eyes with normal health and early AMD were compared with a small sample of young adult eyes to preliminarily assess the effect of age.

## Methods

### Compliance

The Institutional Review Board at the University of Alabama at Birmingham approved this study (protocol #300004907). The study was conducted following the tenets of the Declaration of Helsinki and the Health Insurance Portability and Accountability Act of 1996.

### Study Population

We used baseline data from ALSTAR2 (ClinicalTrials.gov identifier: NCT04112667).^27^^,^^43^ As detailed in the Supplementary Methods, multimodal imaging and visual function data were collected from participants aged 60 years and older recruited from the Callahan Eye Hospital Clinics, between October 2019 and September 2022.^44^ Participants aged 20 to 30 years with normal macular health were enrolled through the same clinics between August and December 2020, meeting the same clinical eligibility criteria as described elsewhere in this article.^45^

### Clinical Image Capture and Quality Analysis

For multimodal imaging, pupils were dilated with 0.5% tropicamide and 2.5% phenylephrine. Imaging included 2WAF, near-infrared reflectance (NIR), and spectral-domain OCT (HRA Spectralis, Heidelberg Engineering, Heidelberg, Germany) as described elsewhere.^46^^,^^47^ A horizontal macular OCT volume scan centered on the fovea was obtained, consisting of 121 B-scans (automatic real time averaging > 9; image quality 29–47) with 60 µm spacing, covering an area of 8.6 × 7.2 mm (30° × 25°). Protocols for 2WAF image capture were described^27^^,^^42^ and are briefly summarized here. The Spectralis investigational MPOD module utilizes confocal scanning laser ophthalmoscopy with blue (λ = 488 nm) and green (λ = 514 nm) laser diodes for AF excitation. Initial camera alignment, illumination, and focus are performed in NIR mode, after which the camera is switched to sequential imaging at 488 nm and followed by 514 nm. Two 30-second movies, each consisting of 140 frames, are captured by alternating between the two excitation wavelengths, using a barrier filter that blocks wavelengths shorter than 560 nm.

OCT and 2WAF image data were reviewed for quality (by A.B.). OCT images were reviewed to exclude eyes with pathologies that distorted the foveal pit, such as large drusen, epiretinal membranes, or macular telangiectasia.^48^ Green and blue AF images were excluded if they exhibited uneven illumination, poor focus, or evidence of floaters or other obstructions.

### Image Processing and Analysis

OCT and 2WAF image data were exported in the .xml file format to ImageJ for postprocessing.^49^ All ImageJ plug-ins used in this study are available at https://sites.imagej.net/CreativeComputation/.

A Cartesian coordinate system to standardize anatomical landmarks before analysis was created using NIR and OCT B-scan images (ImageJ plug-in; ‘Find_Fovea_OCT’).^46^^,^^47^ As described elsewhere,^1^ landmarks for the foveal center and edge of the optic nerve head were defined.^46^^,^^47^ The fovea was centered at the maximal inward rise of the ELM in B-scans through the foveal pit. This represents the central bouquet formed by the longest cone photoreceptors^36^^,^^50^^,^^51^ (also called external fovea in classic neuroanatomy; Fig. 2). The edge of the optic nerve head closest to the fovea was found on the NIR image.

**Figure 3.**
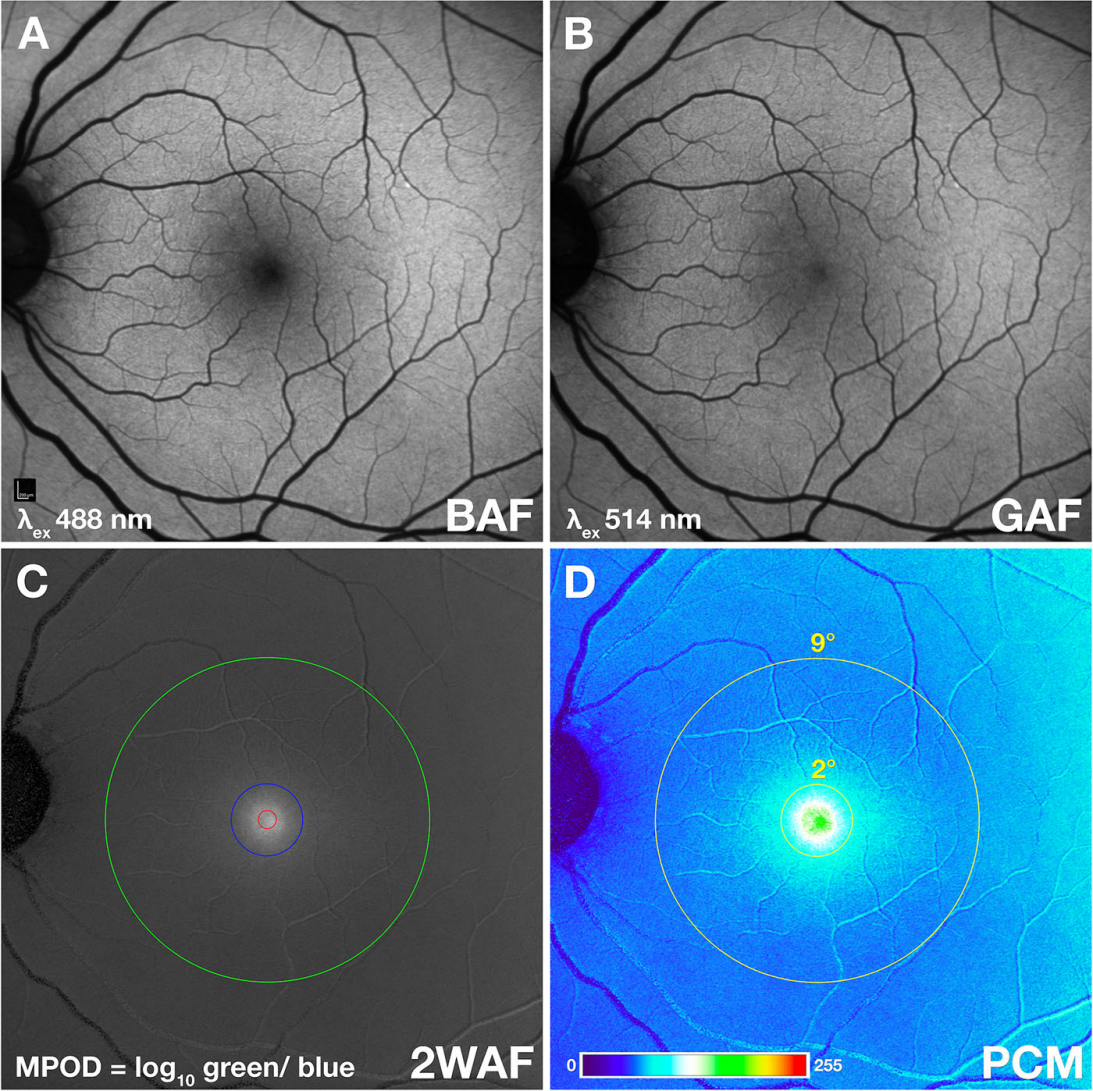
MPOD by 2WAF. (**A**, **B**) MPOD is defined as the log_10_ of the ratio of green-excited AF (GAF) intensities (**B**) to blue-excited AF (BAF) intensities (**A**) emitted by the retinal pigment epithelium, calculated at each pixel. (**C**) From these two input images, a single MPOD image is produced. *Red ring*, 0.5° radius (1° diameter). *Blue ring*, 1° radius (2° diameter). *Green ring*, 9° radius (18° diameter). (**D**) Intensities are displayed in a pseudo color mode (PCM).

Processing of 2WAF image data consisted of four steps. First (Fig. 3), the log_10_ ratio of green-excited to blue-excited AF intensities at each pixel location were used to create 32-bit gray scale and 8-bit pseudocolored MPOD images (MPOD_XML_Reader) for qualitative review.^27^^,^^33^^,^^42^ Second, the 32-bit gray scale image was registered to the coordinate system described elsewhere in this article, resulting in an image stack (‘Register_OCT2’). These two steps, as described,^27^^,^^33^^,^^42^ are required for further analysis. As detailed elsewhere in this article, the third step automatically centers the MPOD distribution (MPOD_Center_OCT) and computes MPOD values. The fourth step automatically assigns the MPOD distribution pattern (MPOD_OCT) into one of the four patterns introduced by Obana et al.^20^ A plugin overview is provided in Supplementary Table 1.

### Automatic MPOD Distribution Centration

**Figure 4.**
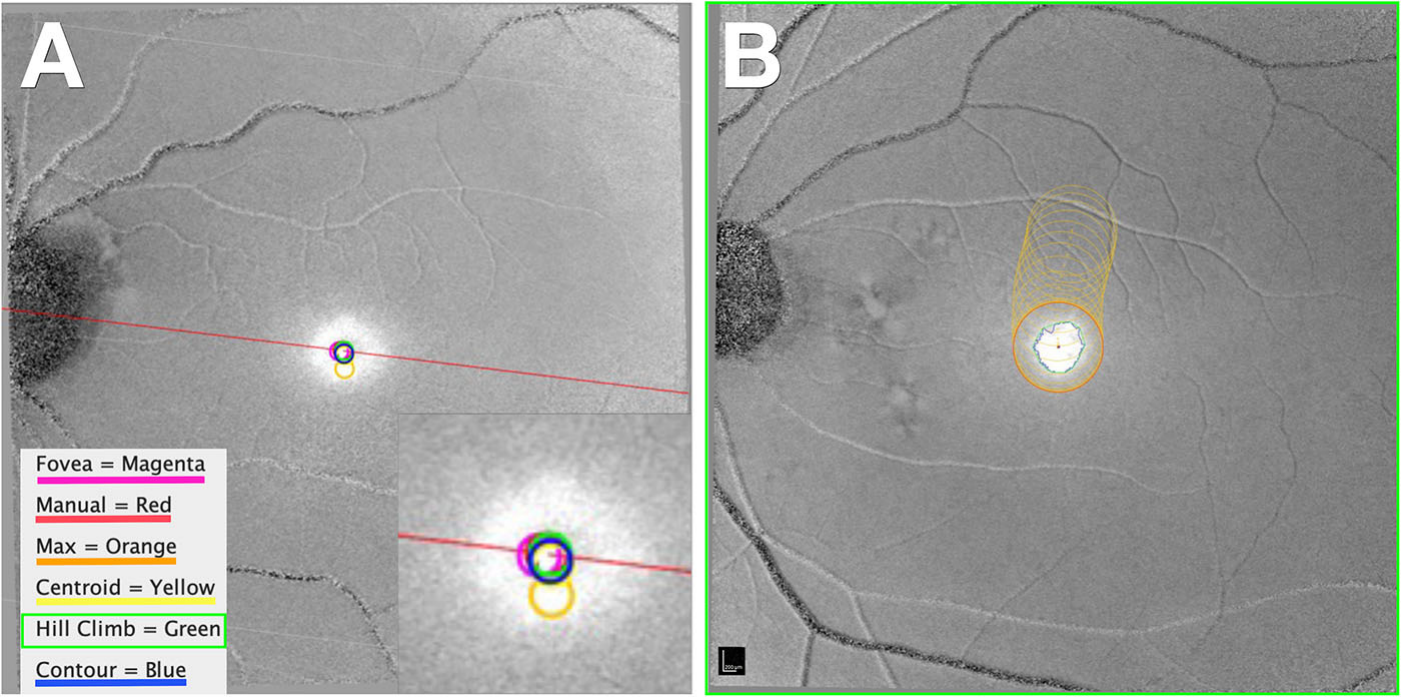
Automatic centration of MPOD distribution. The custom FIJI plugin “MPOD Center OCT” centers the MP distribution using six different techniques: Manual: centered by a trained observer and considered the ground truth. Fovea: Based on the rise of the ELM, independently determined manually by a trained observer using OCT. Max: maximum intensity in a central region. Centroid: weighted average in a central region. Contour: weighted average in the convex hull of a polygon delineating half-maximum intensities. HILLCLIMB: iterative adjustment of the centroid by recomputing until convergence. (**A**) Different centering techniques are highlighted in a magnified inset on the right and legend on the left. (**B**) Automatic centration of the MPOD distribution by HILLCLIMB technique. This iterates a centroid (*orange circle*), adjusts the center, and recomputes a centroid until convergence on the manually defined center is achieved.

To determine the best method for centering the MPOD distribution on OCT-registered en face stacks, the distribution was centered automatically using five methods that were compared with manual centration by a trained observer (A.B.), using a custom FIJI plugin, MPOD_Center_OCT (Fig. 4A). The center of the MPOD distribution was determined manually by a single examiner through subjective visual inspection of both gray scale and false-color mode images. Using the cursor, a crosshair was placed at the center by initially positioning it below the presumed center and then gradually lifting it vertically until it appeared centered (i.e., with approximately equal distances to the edges above and below). The crosshair was then adjusted horizontally and released to set the center. This procedure was repeated two to three times to ensure consistency. A 10% subset of randomly chosen eyes was graded by a second reader (L.G.), and agreement between readers was examined to ensure repeatability.

Automated methods included FOVEA (rise of the ELM on OCT), MAX (maximum MPOD gray value intensity in the central region), CENTROID (a weighted average within the central region), CONTOUR (a weighted average within the convex hull of a polygon delineating half-maximum intensities), and HILLCLIMB (an iterative adjustment of the centroid until convergence). Figure 4B shows how HILLCLIMB iteratively adjusts the center and recalculates a centroid. The plugin can be run with and without manual centration. Users can process single or multiple cases simultaneously. To validate performance and agreement among methods, descriptive statistics of pixel distances from the manually set MPOD center were written to a .tsv file. These values were also used to create scatter plots with 95% prediction ellipses. Pixel distances were converted to µm with a universal factor (11.3 µm/pixel).

### MPOD Distribution Pattern Assignment

**Figure 5.**
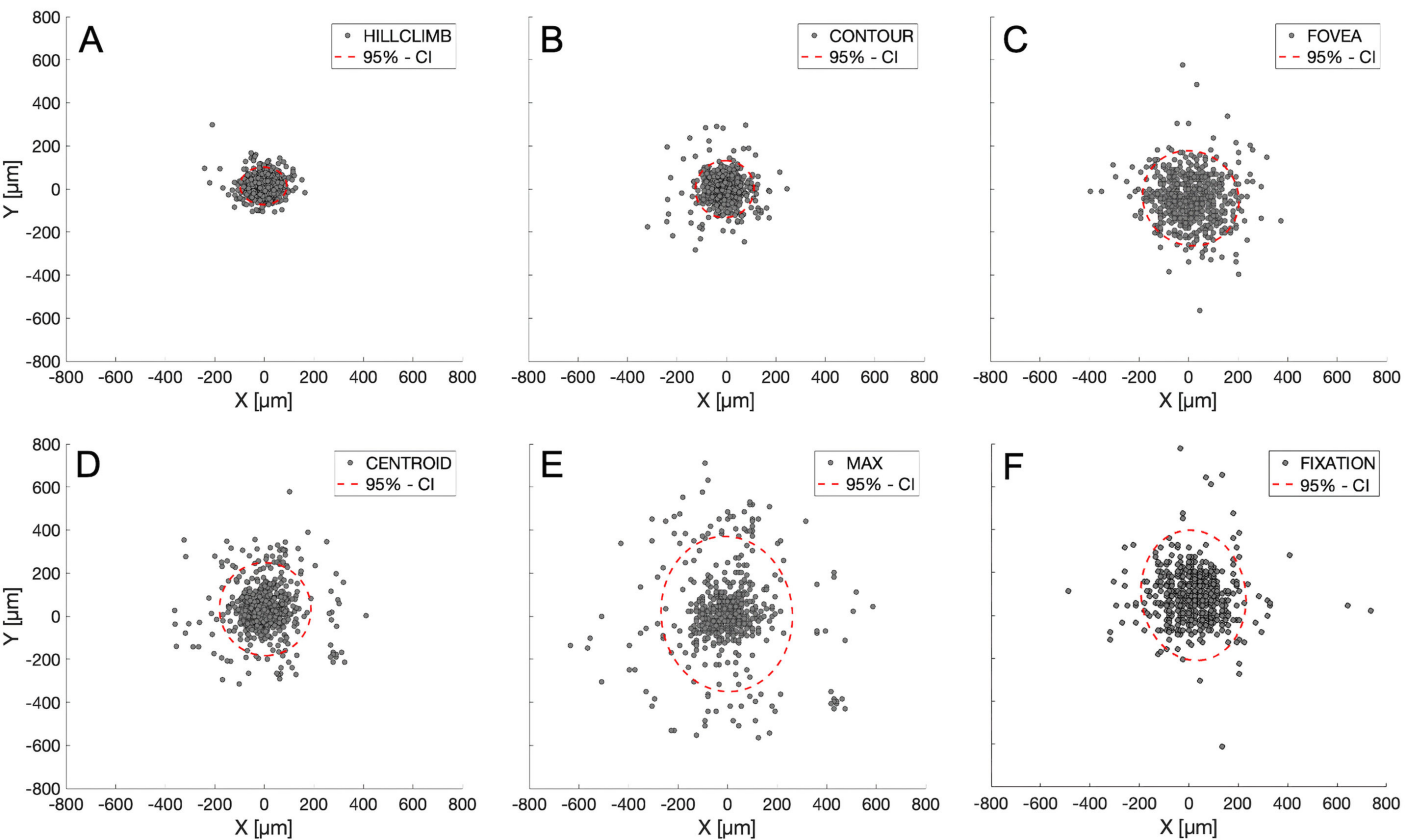
Comparison of different methods for centering the MPOD distribution in 2WAF images. (**A–E**) Five different methods were referenced to manual centration (“MANUAL”) by a single observer. Axes indicate distance from the manually determined center in µm, applying 11.3 µm per pixel. (**A**) Distance of MPOD center defined by the HILLCLIMB method relative to a manually identified center in micrometers. Gray markers represent individual eyes, and the red dotted circle shows the 95% confidence ellipse (CE). The markers are close together, indicating good agreement between the two methods. (**B**) By the CONTOUR method, markers for individual eyes are more spread out compared with (**A**). (**C**) By the FOVEA method, markers are further spread out. (**D**) By the CENTROID method, the 95% CE circle appears similar to (**C**). (**E**) By the MAX method, markers are spread out further. The 95% CE circle is the largest, which indicates limited agreement with the MANUAL method. (**F**) Distances between the center of each image, defined by the indwelling software as the point of fixation and the MANUAL method are qualitatively intermediate between outcomes in (**D** and **E**).

Obana et al.^20^^,^^34^ classified the MPOD distribution into four radially symmetric patterns (Fig. 1) and correlated them to cross-sectional profiles on OCT. In our sample, patterns were assigned automatically to these patterns using a custom FIJI plugin, MPOD_OCT, in two steps. First, the MPOD center is either manually set or determined by running the integrated 'MPOD_center_OCT' plugin (Fig. 5A3). If neither a manually preset center using the 'Find_Fovea' plugin nor an automatically preset center using the 'MPOD_center_OCT' plugin is available, the center must be determined. After setting the MPOD center, the plugin automatically evaluates the MPOD spatial distribution across 12 clock-hour wedges of 30°, with tips at the fovea. The MPOD_OCT plugin generates spatial distribution plots of MPOD intensity and radial extent (Fig. 5B1), with manually modifiable reference lines at 0.5°, 2.0°, and 9.0° eccentricities (Fig. 5A3). Additionally, the plugin offers detailed evaluation within the central 3°(Fig. 5B2) for a refined interpretation of the Obana et al.^20^ patterns. The MPOD pattern in each 30° wedge is classified into one of four types with simplified names (peak, ring, mixed, or dip; Fig. 5B3). The results of the automatic assessment are displayed, and the overall pattern for each eye is determined by a simple majority vote across the 12 wedges (7 out of 12) (Fig. 5B3). Users have the option to manually override the classification of individual wedges, the overall pattern, or both (Fig. 5B3). Assignments made by both the plugin and the user for each 30° wedge and the overall MPOD pattern are written to a .tsv file. Then, MPOD values are computed in three different geometric categories: mean MPOD measured on radial circles (ALL) from 0° to 9° eccentricity, mean MPOD measured on radial discs (VOLUMES) from 0° to 9° eccentricity, and mean MPOD measured on 30° bowtie wedges (WEDGES) within 9° of eccentricity around the center. To streamline analysis, the MPOD_OCT plugin allows users to assess single or multiple cases simultaneously (Fig. 5A3).

### Statistical Analysis

Data on sample demographics, AMD severity, MPOD centration, and MPOD patterns were analyzed at eye level. AMD status was classified as normal (AREDS 1) or early (AREDS 2–4). For the five automatic MPOD centration methods, distances from the manually assigned center were recorded, with the shortest mean distance considered the best match. Frequencies and intraclass correlation coefficients between the manual grader and the software were reported for the four patterns described elsewhere in this article.^20^ Associations between MPOD distribution patterns and demographic characteristics were analyzed using a generalized estimating equation approach. Agreement analysis between the readers and between the readers and the plugin was conducted. For MPOD centration, visual inspection of scatter plots comparing the *x* and *y* coordinate values, Bland–Altman plots, calculations of Euclidean distance between the *x* and *y* coordinates, and Cohen's kappa statistics were performed. For MPOD pattern classification, the intraclass correlation coefficient was computed. All analyses were performed using SAS (Version 9.4; SAS Institute Inc., 2025). A *P* value of 0.05 of less was considered significant.

**Figure 6.**
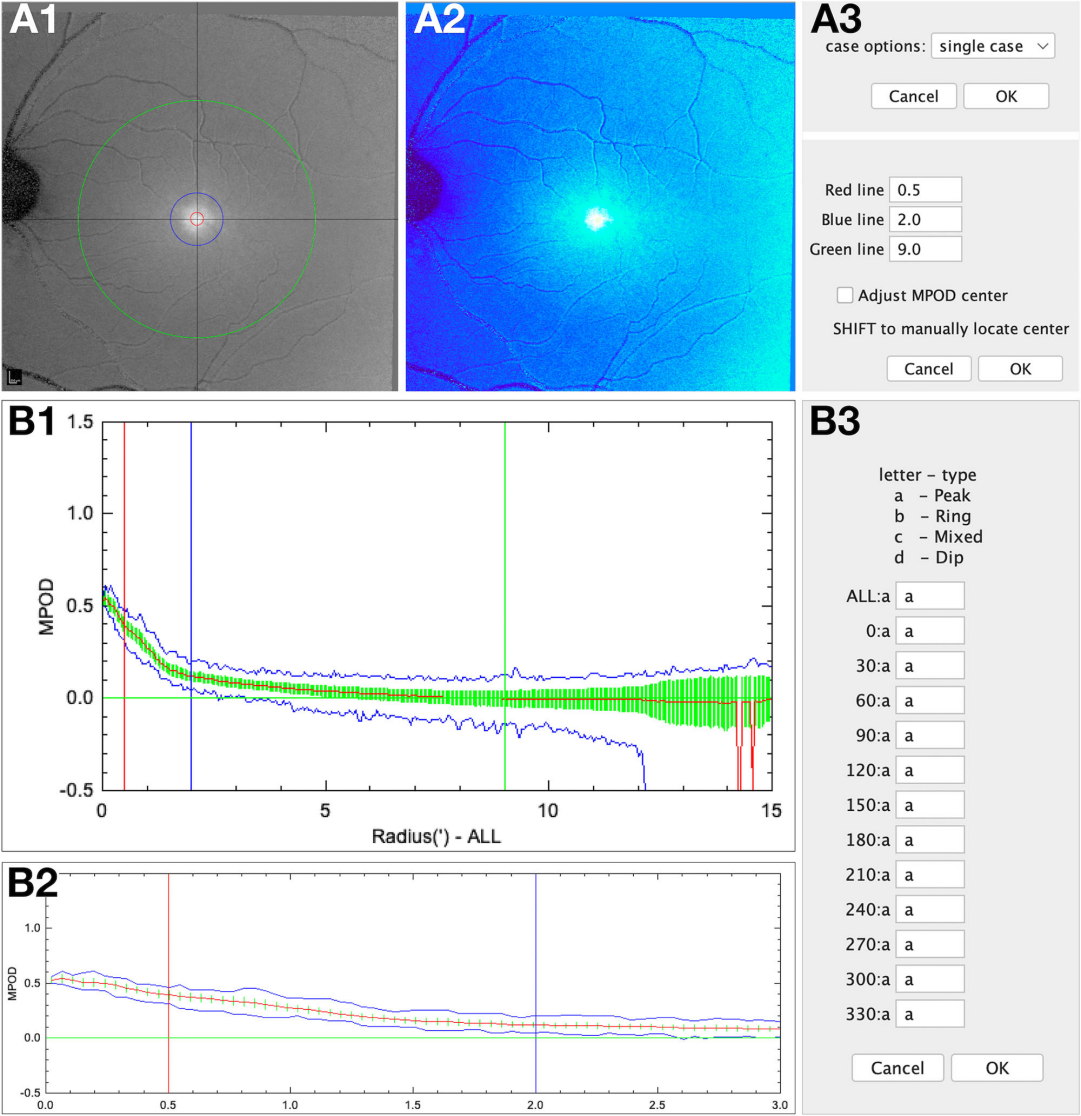
Automatic assignment of MPOD distribution pattern. The FIJI plugin MPOD OCT assigns the pattern at each of 12 clock-hours into 4 types of radial symmetry: peak, ring, mixed, and dip (Fig. 3), according to Obana et al.,^20^ who used a, b, c, and d (Fig. 5B3), for these patterns, respectively. (**A**) Gray scale image (**A1**) and pseudocolor mode image (**A2**) of MPOD are displayed. Interface for case options and plot parameters (**A3**). Users can process single or multiple cases simultaneously and set the center of the MPOD distribution manually. Plot parameters include options for measuring MPOD at different eccentricities: *red line* (0.5°), *blue line* (2°), and *green line* (9°, measurement reference). (**B**) Spatial distribution plot (**B1**) showing MPOD intensity (*x* axis) and radial extent (*y* axis). This includes 12 plots (not shown) representing 30° wedges, along with a combined plot of all 12 wedges (**A1**). Detailed analysis (**B2**) of the central 3° for enhanced evaluation and interpretation according to Obana et al.^20^ Results from the automatic assessment are displayed (**B3**), which can be manually adjusted if it does not match the manual assignment. The 13 automatic results, as well as the manually approved assessments, are stored in a .csv tab file.

Figure 6 (scatter plot of assigned centers) was created using MatLab (Version 9.5; The MathWorks, Natick, MA, USA; code downloadable at: https://www.mathworks.com/matlabcentral/fileexchange/26311-raacampbell-shadederrorbar).

## Results

Of 887 normal and early AMD eyes from the ALSTAR2 baseline cohort, 227 were excluded owing to the unavailability of MPOD images, insufficient image quality, or pathologies distorting the foveal pit (Supplementary Table 2). Thus, 651 eyes of 370 individuals (mean age, 71.2 ± 5.8 years; 408 female eyes [62.7%]) (Table 1) were used for MPOD centration and distribution pattern analysis. Among 50 young eyes of 25 young individuals, 6 were excluded owing to missing images, leaving 44 young eyes of 23 individuals (mean age, 25.0 ± 3.3 years; 27 female eyes [61.4%]) (Table 1) for MPOD distribution pattern analysis. Normal (*n* = 427) and early AMD (*n* = 224) eyes are shown in Table 2. Nearly two-thirds of normal eyes were phakic, whereas early AMD eyes were equally divided between phakic and pseudophakic. In 55 eyes (8.4%, Table 1), supplementation with lutein, zeaxanthin, and vitamins was reported.

**Table 1.** Demographic Characteristics of 370 ALSTAR2 Participants

Characteristic	Value
Age (years)	71.3 ± 6.0
Age group	
60–69	142 (38.4)
70–79	197 (53.2)
80–89	29 (7.8)
90–99	2 (0.5)
Gender	
Female	228 (61.6)
Male	142 (38.4)
Race	
White	327 (88.4)
African American	35 (9.5)
Asian or Pacific Islander	8 (2.2)
Lens status	
Phakic	362 (55.6)
Pseudophakic	289 (44.4)
Supplement use*	
Yes	55 (8.4)
No	601 (92.6)

Values are mean ± standard deviation or number (%).

Young controls (*n* = 23): age 25.0 ± 3.3 years; gender (female, *n* = 14 [60.9%]; male *n* = 9 [39.1%]), race (White, *n* = 20 [87.0%]; African American, *n* = 3 [13.0%]).

* Self-reported supplementation with lutein and/or zeaxanthin such as Preservision/Age-related Eye Disease Study (AREDS), Lutigold, Provision, Macular Health, Adult 50+ Eye Health, Ocuvite.

**Table 2.** The 651 Study Eyes Stratified by AREDS Presence and Aeverity

	Normal	Early AMD
No. of eyes	427	224
Age, years	71.0 ± 5.9	71.5 ± 5.8
Gender		
Female	281 (65.8)	127 (56.7)
Male	146 (34.2)	97 (43.3)
Lens status		
Phakic	251 (58.8)	111 (49.6)
Pseudophakic	176 (41.2)	113 (50.4)

SD, standard deviation.

Agreement between the two readers was generally acceptable (Supplementary Fig. 1). The mean difference in overall agreement between the graders (L.G. and A.B.) was 9.41 pixels, calculated using the Euclidean distance between their *x* and *y* coordinate values, indicating acceptable agreement. The intraclass correlation coefficient for *x* coordinates was 0.53, reflecting fair to moderate agreement, while the intraclass correlation coefficient for *y* coordinates was 0.39, indicating poor to fair agreement. Overall, these findings demonstrate moderate but sufficient consistency between graders in the context of en face imaging of gray value images, but also underscore the need for improved consistency in future assessments.

In 651 aged eyes, five automated methods for MPOD centration were compared with manual centration (A.B.) (Fig. 5; Table 3). As assessed by mean distance to MANUAL (55.3 ± 35.9 µm and 70.4 ± 54.6 µm, respectively; *P* < 0.01) HILLCLIMB and CONTOUR demonstrated the best overall agreement with MANUAL ground truth. In contrast, CENTROID, FOVEA, and MAX exhibited worse overall agreement (in ascending order of mean distances to MANUAL, 111.4 ± 92.3 µm, 127.1 ± 85.9 µm, and 161.4 ± 155.5 µm, respectively; *P* < 0.01).

**Table 3.** Automatic Centration of the MP Distribution

Distance in µm*	HILLCLIMB	CONTOUR	FOVEA	CENTROID	MAX	Overall P-Value
Minimum	1.6	3.2	0.00	3.4	0.00	<0.01
Mean	55.3	70.4	127.1	111.4	161.4	
Maximum	364.9	363.2	576.8	587.6	717.7	
SD	35.9	54.6	85.9	92.3	155.5	

Radial distances in pixels between MANUAL reference centration and five automatic methods of MPOD Center OCT in 651 eyes.

* Conversion factor 11.3 µm/pixel.

Differences among methods are clearly visible in scatter plots of automated vs. manual centers for each eye, as 95% confidence ellipses expand as centration quality worsens. Of note, a foveal center assigned to NIR images by the Spectralis device at the point of fixation is intermediate between CENTROID and MAX in approximating MANUAL centration (Fig. 5E).

Results of automatically assigning MPOD distribution patterns are summarized in Table 4. In 651 aged participants, peak was dominant (445 eyes [68.4%]), followed by ring (118 eyes [18.1%]). Mixed and dip were the least common, with similar frequencies (41 eyes [6.3%] and 47 eyes [7.2%], respectively). Agreement between automated and manual classifications was strong across the entire dataset of 651 samples, with a simple kappa statistic of 0.831 ± 0.022 (range, 0.788–0.874). Agreement between two readers was moderate with a simple kappa statistic 0.473 ± 0.119 (range, 0.240–0.707). Among young participants, the contrast among the four types was even more pronounced. In 90.9% of young eyes, a peak was observed. Rings and mixed patterns were rare (1 eye [2.3%] and 3 eyes [6.8%], respectively). The dip pattern was not observed.

**Table 4.** Frequency Distribution of MPOD Patterns by Age

Distribution Pattern	N (%)
651 Aged eyes*	
Peak	445 (68.4)
Ring	118 (18.1)
Mixed	41 (06.3)
Dip	47 (07.2)
44 Young eyes**	
Peak	40 (90.9)
Ring	1 (02.3)
Mixed	3 (06.8)
Dip	0 (00.0)

For each eye, the MPOD distribution derived from 12 clock-hour wedges was classified into one of four types (Peak, Ring, Mixed, or Dip) based on majority vote and manually corrected if needed. In aged participants, the Peak type was predominant, followed by the Ring pattern. Mixed and Dip were the least common, with similar low frequencies. Among young participants, the contrast among the four types was even more pronounced. In over 90% of eyes, a Peak type was observed. Ring and Mixed were rare, while the Dips were entirely absent in these young eyes.

* 651 aged eyes (normal and early AMD, see Table 2 for details) without pathology affecting the foveal shape.

** See Table 1 for details.

Table 5 shows the frequency distribution of MPOD patterns by AMD severity. In normal and early AMD eyes, peaks were the most prevalent pattern (67.6% and 69.6%, respectively), followed by rings (19.0% and 16.5%, respectively). Mixed and dip types were the least common, each occurring at similarly low frequencies (5.4% vs. 8.0% and 8.0% vs. 5.8%, for normal and early AMD, respectively).

**Table 5.** Association of MPOD Pattern With Demographic Characteristics

	Obana et al. Pattern									
Characteristic	No.	Peak		Ring		Mixed		Dip		*P* Value
Age group, years										
** **60–69	251	184	73.3%	44	17.5%	9	3.6%	14	5.6%	
** **70–79	348	230	66.1%	61	17.5%	28	8.0%	29	8.3%	0.14
** **80–99	52	31	59.6%	13	25.0%	4	7.7%	4	7.7%	
Gender										
Female	408	265	65.0%	90	22.1%	23	5.6%	30	7.4%	
Male	243	180	74.1%	28	11.5%	18	7.4%	17	7.0%	0.03
Race										
White	575	406	70.6%	95	16.5%	34	5.9%	40	7.0%	
African American or other	76	39	51.3%	23	30.3%	7	9.2%	7	9.2%	0.13
Lens status										
Phakic	362	235	64.9%	64	17.7%	31	8.6%	32	8.8%	
Pseudophakic	289	210	72.7%	54	18.7%	10	3.5%	15	5.2%	0.04
AREDS grade										
Normal	427	289	67.7%	81	19.0%	23	5.4%	34	8.0%	
Early AMD	224	156	69.6%	37	16.5%	18	8.0%	13	5.8%	0.45

Persons with pseudophakic eyes were significantly older than persons with phakic eyes (69.5 ± 5.2 vs. 73.3 ± 5.8 years; *P* < 0.01).

**Figure 7.**
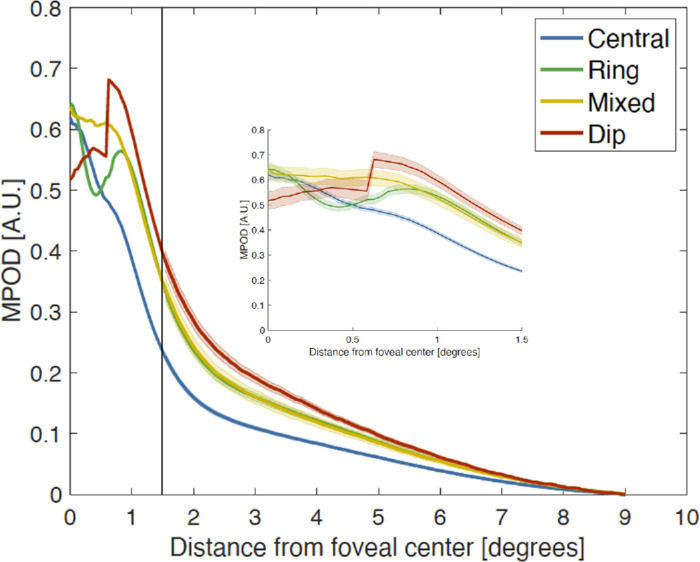
Spatial profiles of four different distribution patterns of MP in the ALSTAR2 cohort. Spatial MPOD profiles with error bands (standard error) along the horizontal meridian reflect the different appearances of four distribution patterns, per Obana et al.,^20^ in the en face view of the MPOD images of 651 study eyes. “Peak” was the most common MP distribution pattern (445 eyes [68.4%]), followed by Ring (118 eyes [18.1%]). Mixed and Dip patterns were the least frequent, with similar distributions (41 eyes [6.3%] and 47 eyes [7.2%], respectively). The central 1.5° is magnified in the inset. The tight error bands for central reflects the large number of eyes with this type. MPOD in foveas with dips is lower than that of the other types, as highlighted in the magnified inset on the right. MPOD in foveas with rings was lower at 0.5° than those with Mixed. MPOD in foveas with peaks was lower at 0.5° than those with Mixed. The MPOD of Peaks at 0.5° to 1.9° was lower than that of the rings and mixed. Finally, MPOD of Peaks at 2.0° to 4.0° was lower than that of the other three patterns.

Graphic representation of MPOD patterns in the 651 older participants (Fig. 7) highlights the variability in MPOD spatial distribution. Individuals with dips had the lowest amount of foveal MPOD with the most pronounced reduction occurring in the central-most region. Those with peaks had the lowest total MPOD across the Early Treatment Diabetic Retinopathy Study central subfield (inset of Fig. 7). In eyes with rings, the MPOD at 0.5° eccentricity was lower compared with those with mixed and dip patterns. Similarly, eyes classified as peaks also exhibit lower MPOD from 0.5° to 1.9° eccentricity than those with mixed or dip patterns (Fig. 7).

Associations between MPOD distribution patterns and demographic characteristics are shown in Table 5. No significant differences were observed among age ranges older than 60 years (*P* = 0.145), race (*P* < 0.13), or AREDS grade (*P* = 0.368). Significantly fewer peaks and more rings (*P* = 0.008) were found in females (65.0% and 22.1%, respectively) compared with males (74.1% and 11.5%, respectively), whereas mixed and dip patterns were evenly distributed. Lens status was associated with MPOD patterns (*P* = 0.011), with phakic eyes showing proportionately fewer peaks (64.9%) than pseudophakic eyes (72.7%). The proportion of women and men with phakic eyes did not differ (219 [53.68%] vs. 143 [58.85%], respectively; *P* < 0.20, χ^2^ test). However, persons with pseudophakic eyes were significantly older than persons with phakic eyes (73.3 ± 5.8 vs. 69.5 ± 5.2 years; *P* < 0.01). Because women may have neovascular AMD more frequently than men,^52^ we asked if MPOD patterns were associated with disease stage if stratified by gender (Supplementary Table 3)*.* Peak is the most common pattern, and no significant associations were found.

## Discussion

Imaging with 2WAF is a highly reproducible metabolic imaging approach to cells of the high-risk macula lutea.^34^^,^^53^^–^^55^ Herein we present advanced analysis tools to automatically center the MPOD distribution and assign one of four previously described MPOD patterns. These tools can be used to probe foveal metabolism, the aging process, and AMD pathophysiology. Our data suggest that the MPOD spatial distribution differs prominently between women and men and between pseudophakic vs. phakic eyes. Because a recent large study indicates that oral supplementation with MXCs can slow the expansion of geographic atrophy in AMD,^56^ our new tools are a timely addition to the AMD armamentarium for monitoring supplementation and diet effects in future studies.

Our tools incorporate a cellular basis of MPOD that has been refined in three ways. First, Müller glia in the macula lutea are major MXC reservoirs and molecularly distinct from all other Müller glia.^57^^,^^58^ In tissue assays, visualized MXCs are concentrated at the foveal center, including the outer nuclear, plexiform, and nerve fiber layers,^9^^,^^59^^,^^60^ but not in outer segments, as suggested by extractions.^61^^,^^62^ Further, MXCs and Müller glial molecular markers disappear together in macular telangiectasia^63^^,^^64^ and appear together in surgically excised proliferative membranes.^65^^,^^66^ Glial MXC helps to explain why MXC appear in inner retinal layers that lack photoreceptors, yet does not exclude contributions from other inner retinal cells. Second, variation in MPOD is related to foveal dimensions and shape,^61^ which are in turn related to a precisely orchestrated prenatal and postnatal development sequence.^50^^,^^62^^,^^63^ This includes centrifugal migration of inner retinal neurons from the foveal pit, squeezing of cone inner segments into the foveal center, and elongation of photoreceptor axons to maintain contact with postreceptoral neurons.^36^^,^^65^ Inner retinal transformations begin earlier and proceed independent of those in outer retina. Third, when viewed in 2WAF images, variation in the MXC distribution leads to alternating rings of higher and lower intensities distributed around a center point.^20^^,^^33^^,^^42^ The *z* score maps that converted absolute pixel level data from groups of eyes into units of standard deviation of the entire sample revealed annuli of higher and lower than average MPOD, following the distribution of MXC-containing cells in retinal layers.^54^

One new tool, MPOD_center_OCT, provides standardized and automated centration of the MPOD distribution.^17^^,^^18^^,^^20^ This plugin can be compared with other efforts to define and automate localization of an anatomical foveal center. Structural OCT as deployed clinically defines a center as the minimum distance between the internal limiting membrane and the retinal pigment epithelium–Bruch's membrane within the foveal pit.^67^ Another OCT approach considers the maximum distance between the ellipsoid and interdigitation zones, capturing the outer segment. Unlike these methods, we centered the MPOD separate from an anatomical landmark (inward rise of the ELM). For reference, adaptive optics scanning laser ophthalmoscopy and OCT center the fovea using a functional attribute, the preferred retinal locus (point of visual fixation).^68^ Relative to manual assignment of the MPOD distribution, the fixation point was only intermediate in accuracy in our sample.

Our centration algorithm achieves a precision of 55.3 ± 34.9 µm, compared with CFP^69^^–^^75^ (7.39 ± 5.54 µm^71^ to a median of 199 µm^75^) and OCT (54 ± 41 µm^76^ to 104 ± 62 µm).^76^^,^^77^ This level of interobserver and intermethod variability highlights the challenges in localizing a center consistently. Specifically, centration of CFP used in studies of geographic atrophy in AMD may have a localization error of 178 to 199 µm^69,75^ diameter (0.61°–0.69° in an average eye^78^). These values exceed the estimated diameter of the central bouquet and fall within the region of highly variable MPOD revealed by meridian plots^20^ (0.6° diameter, 0.3° radius) (Fig. 7). Precision is critical for studies involving foveal shape as sculpted by developmental processes,^79^^–^^81^ MXC bioavailability, and AMD risk. The spread of MPOD centers around the ELM increase on OCT (by the FOVEA method) (Fig. 5C) supports our approach. Thus, we will continue using HILLCLIMB and CONTOUR.

To our knowledge, this study is the first to automate assessment of the MPOD distribution pattern. The variable shape of this distribution, now captured by imaging,^17^^,^^18^^,^^20^^,^^29^^,^^54^ was apparent even in psychophysical testing at select retinal locations.^82^^,^^83^ To date, MPOD distribution patterns have been classified manually, which is subjective and time consuming, relying on expert evaluation and interobserver agreement.^17^^,^^18^^,^^20^^,^^84^ By plotting the eccentricity relationship of MPOD across eyes (Fig. 6), we demonstrated that eyes with peaks have lower, and eyes with rings have higher, MPOD than other patterns. We found equally high values (0.62–0.65 AU) at 0° eccentricity for peak, ring, and mixed types, whereas the equally high dip type (0.69 AU) is maximal at approximately 1° eccentricity. Beyond 0.5° eccentricity, peaks show the lowest MPOD values; other types resemble each other. Our findings are almost identical to those reported by Obana et al. (their Fig. 4),^20^ except that our eyes with rings have higher values at 1°. This difference is possibly due to characteristics of the patient population including larger size, or differences in analysis.

Our data from young adult eyes are limited yet suggest an age difference in MPOD distribution patterns. Peaks were observed in 90.9% of young eyes and in only 59.6% in persons aged 80 to 99 years. Conversely, older individuals more frequently exhibited ring and dip patterns than young eyes (18.1% vs. 2.3% and 7.3% vs. 0%, respectively). Studies using fundus reflectometry imaging have reported similar trends, with younger eyes displaying narrow MPOD profiles, and broader profiles in middle-aged and older eyes.^85^ In 30 eyes in persons 23 to 77 years (70% female, 76% Caucasian), MPOD seen by 2WAF was found to increase to a plateau at 45 to 50 years with a slight decline at older ages.^86^ Whether MXC accumulation, redistribution, or degradation changes throughout adulthood needs further study in a large population with a wide and balanced age range.

Our findings that more women exhibited the ring pattern than men (22.1% vs. 11.5%) add to literature indicating a gender difference in MPOD distribution. Men in our cohort were more likely to have peak patterns (74.1% vs. 65.0%). No differences between women and men were found in aged persons assessed for mean MPOD in the central subfield (France)^84^ or by comparing MPOD at selected eccentricities (Japan).^20^ Our results confirm those from a prototype 2WAF instrument by Delori et al.,^18^ who observed that women exhibit a prominent ring owing to both higher eccentric MPOD and a peripheral distribution, particularly in older individuals. These findings were attributed by the authors to differences in foveal depression size and shape, impacting the distribution of MXCs in the different retinal layers.^9^ This, along with the broader MP distribution in women, suggests that a wider foveal depression contributes to a high frequency of rings.^18^ Structural differences in foveal architecture available from OCT analysis support this conclusion. Wagner-Schuman et al. documented variations in foveal pit depth, width, and thickness in 180 adults^87^^–^^89^ corrected for axial length and found that men have thicker foveas and shallower foveal pits than women. Further, analysis of more than 180,000 OCT volumes from the UK Biobank^90^ revealed that men exhibit steeper foveal curvature and greater central retinal thickness than women. Wagner-Schuman et al^87^ hypothesized the existence of anatomical risk factors like foveal shape for the onset of AMD in late adulthood. Our group expanded this concept by speculating that shape impacts the geometry of MXC delivery from circulation to the neurosensory retina and thus the development of high-risk drusen under the fovea.^27^^,^^42^ These ideas can be investigated further using these new automated analysis tools plus our recently developed visualization tool that incorporates foveal variability into maps.^54^

Our data suggest a potential association between MPOD distribution and lens status. Compared with pseudophakic individuals, phakic individuals were more likely to exhibit mixed patterns (8.6% vs. 3.5%) and less likely to exhibit peak patterns (64.9% vs. 72.7%), and, on average, these persons were 4 years younger (69.5 years vs. 73.3 years). In an older cohort (mean age, 82 years), Alassane et al.^84^ reported a slightly higher prevalence of peaks (referred to as no ring) in phakic compared with pseudophakic eyes (74.7% vs. 70.3%). Optical media alterations such as advanced cataracts and advanced posterior capsule opacification can impact MPOD measurements^91^^–^^93^ primarily by lowering blue AF signal via absorption, of importance if MPOD is an outcome measure for a supplement trial.^94^^,^^95^ Efforts to overcome these alterations^91^^–^^93^^,^^96^ have achieved reasonable compensation yet still show errors in MPOD measurements at low eccentricities and in cases of poor image quality. Nonetheless, optical media alterations primarily affect the total MPOD level rather than its spatial distribution. Thus, a global correction approach for lens absorption is unlikely to influence the MPOD distribution patterns observed in our study. Conversely including eyes with mild cataract for mechanistic studies of foveal shape may be acceptable.

MPOD patterns from individuals of African and Asian descent and from eyes at different AREDS stages did not show significant associations in our study. The frequency of ring patterns in the literature range from 17.2% to 68% in White individuals,^84^^,^^97^^–^^99^ 58% in South Asian individuals, 24% to 86% in Black populations,^99^^,^^100^ and 40.6% in a Japanese cohort.^20^ Obana et al.^20^ reported dips in 12.5% of aged Japanese individuals, slightly higher than in our cohort of mostly European descent. Ctori et al.,^100^ using a heterochromatic flicker photometry, reported frequent dips (17 eyes, (23%) in White and 32 eyes (43%) in African-descent individuals). More dips were observed in older White smokers (18.8%), suggesting that this pattern is associated with AMD risk. In addition to foveal anatomy (as discussed elsewhere in this article), genetics, dietary, and lifestyle differences among Western, African, and Asian populations contribute to differences in MPOD distribution.^101^

A key strength of our study is a comprehensive imaging analysis pipeline for MPOD centration and distribution pattern assessment, in a very large number of eyes. Our analysis tools incorporate a refined cellular basis for MPOD and allow MPOD to be treated as a continuous variable for robust statistical analysis. The inclusion of both phakic and pseudophakic eyes indicate differences that should be investigated further.

Limitations include the single-center study design, predominance of one ethnic group, lack of information about intraocular lens types, and the relatively small number of young adult participants. Moreover, the usefulness of the current analysis tools may be limited in the presence of macular pathology associated with more advanced stages of AMD. Although our focus in this study was on aging and early AMD eyes to help identify individuals at risk for aggressive disease progression, future applications will need to address broader clinical variability. In addition, total MP optical volume was not evaluated here, but remains a promising area for future investigation, particularly in light of potential confounding factors such as age-related cataracts. Although inter-rater agreement for MPOD centration and pattern distribution was moderate, this level of agreement is comparable with early experiences with other image grading systems, such as color fundus photography.^102^ Importantly, even with moderate agreement, valuable insights can be gained, fostering continuous improvement in emerging technologies like 2WAF imaging.^103^^–^^105^ Future efforts should focus on refining grading protocols and training procedures to enhance consistency between graders and further advance this promising technology.

Future studies within the ALSTAR2 cohort will leverage our novel tools to explore key aspects of MXC bioavailability. This includes the impact of foveal shape on MPOD patterns, the relationship between MPOD and cone- and rod-mediated vision, and the connection between MPOD and dietary xanthophyll content, as regulated by the high-density lipoprotein pathway of AMD genetics.^39^ The integration of machine learning approaches will refine automated centration and classification systems, enhancing diagnostic and prognostic accuracy and monitoring the impact of MXC in AMD and other retinal degenerations involving the fovea.^106^^,^^107^ It also means that 2WAF may assist in assessing conditions that affect foveal Müller glia. Finally, spectroscopic information from 2WAF may be used to enrich structural imaging through transfer learning to OCT volumes of the same individuals.

## Supplementary Material

Supplement 1

Supplement 2

Supplement 3

Supplement 4

Supplement 5
