# Supplementary material for: Advanced Analysis Tools for Two Wavelength Autofluorescence Imaging of Macular Xanthophyll Carotenoids: ALSTAR2 Baseline
Source: Transl Vis Sci Technol. 2025 Aug 21;14(8):32. doi: 10.1167/tvst.14.8.32 (PMC12393178; doi:10.1167/tvst.14.8.32)
Supplement: Supplement 1 [file tvst-14-8-32_s001.docx]

**Supplementary Methods**

Multimodal imaging and visual function data were collected from participants aged 60 years and older recruited from the Callahan Eye Hospital Clinics, the clinical service of the University of Alabama at Birmingham Department of Ophthalmology and Visual Sciences. Participants were identified by searching electronic medical records for ICD-10 codes of early AMD and intermediate AMD (H35.30*, H35.31*, H35.36*). Medical records were manually screened to confirm eligibility (author C.O.). Exclusion criteria included prior diagnoses of glaucoma, other retinal or optic nerve conditions, corneal disease, refractive error greater than 6 diopters, diabetes, Alzheimer’s disease, Parkinson’s disease, brain injury, or other neurological or psychiatric conditions, as identified through medical records or self-report by participants. Demographic information was obtained via self-administered questionnaires. Eyes were assigned AMD status using three-field digital stereo color fundus photographs (Carl Zeiss Meditec 450+, Dublin, CA) by an experienced, masked grader (author MEC) following the AREDS 9-step classification system.^1^ Repeatability statistics for this method have been reported.^2^ The AREDS classification has a strong epidemiological basis using a classification and regression tree analysis^3^ and was used for a power analysis in the initial design of ALSTAR2.^1, 4^ This report included eyes in normal macular health (AREDS grade 1) or classified as early AMD (AREDS grades 2–4).

References:

1. Davis MD, Gangnon RE, Lee LY, et al. The Age-Related Eye Disease Study severity scale for age-related macular degeneration: AREDS report No. 17. *Archives of ophthalmology (Chicago, Ill: 1960)* 2005;123:1484-1498.

2. Echols BS, Clark ME, Swain TA, et al. Hyperreflective Foci and Specks Are Associated with Delayed Rod-Mediated Dark Adaptation in Nonneovascular Age-Related Macular Degeneration. *Ophthalmology Retina* 2020;4:1059-1068.

3. Chew EY, Peto T, Clemons TE, et al. Macular Telangiectasia Type 2: A classification system using multimodal imaging MacTel project report number 10. *Ophthalmology Science* 2023;3:100261.

4. Curcio CA, McGwin G, Sadda SR, et al. Functionally validated imaging endpoints in the Alabama study on early age-related macular degeneration 2 (ALSTAR2): design and methods. *BMC ophthalmology* 2020;20:1-17.
