# Supplementary material for: Advanced Analysis Tools for Two Wavelength Autofluorescence Imaging of Macular Xanthophyll Carotenoids: ALSTAR2 Baseline
Source: Transl Vis Sci Technol. 2025 Aug 21;14(8):32. doi: 10.1167/tvst.14.8.32 (PMC12393178; doi:10.1167/tvst.14.8.32)
Supplement: Supplement 2 [file tvst-14-8-32_s002.docx]

**Supplementary Table 1. Pipeline of ImageJ plugins used for automated MPOD centration and MPOD distribution assignment**

| **Plug-in name** | **Function** |
| --- | --- |
| Find_Fovea_OCT | Creating Cartesian coordinate system. |
| MPOD_XML_Reader | Creates an MPOD image from MPOD XML Export |
| Register_OCT2 | Registers en face images to OCT XML Export, creating an image stack. |
| MPOD_center_OCT | Automatic centration of the MPOD distribution independent of the external fovea (rise of the ELM) using 5 different approaches: FOVEA, MAX, CENTROID, CONTOUR, and HILLCLIMB. |
| MPOD_OCT | Automatic, simple-majority based assignment of MPOD distribution to one of 4 patterns according to Obana et al: Peak, Ring, Mixed, and Dip.^1^ |

**References:**

1. Obana A, Gohto Y, Sasano H, et al. Spatial distribution of macular pigment estimated by autofluorescence imaging in elderly Japanese individuals. *Japanese journal of Ophthalmology* 2020;64:160-170.
