# Supplementary material for: Advanced Analysis Tools for Two Wavelength Autofluorescence Imaging of Macular Xanthophyll Carotenoids: ALSTAR2 Baseline
Source: Transl Vis Sci Technol. 2025 Aug 21;14(8):32. doi: 10.1167/tvst.14.8.32 (PMC12393178; doi:10.1167/tvst.14.8.32)
Supplement: Supplement 3 [file tvst-14-8-32_s003.docx]

| Exclusion criteria stratified by  AREDS classification | | | Value | |
| --- | --- | --- | --- | --- |
|  |  | | |  |
| Eyes excluded | | | 227 | (100.0%) |
|  | | |  |  |
| Normal total | | | 109 | (48.0%) |
| No images available | | | 44 | (19.4%) |
| Insufficient image quality | | | 26 | (11.5%) |
| Distorted fovea | | | 39 | (17.1%) |
|  | | |  |  |
| Early AMD | | 118 | | (52.0%) |
| No images available | | | 36 | (15.9%) |
| Insufficient image quality | | | 31 | (13.7%) |
| Distorted fovea | | | 51 | (22.4%) |
|  | | |  |  |

## **Supplementary Table 2. Overview of 227 aged eyes excluded from final analysis**
