# Supplementary material for: Advanced Analysis Tools for Two Wavelength Autofluorescence Imaging of Macular Xanthophyll Carotenoids: ALSTAR2 Baseline
Source: Transl Vis Sci Technol. 2025 Aug 21;14(8):32. doi: 10.1167/tvst.14.8.32 (PMC12393178; doi:10.1167/tvst.14.8.32)
Supplement: Supplement 4 [file tvst-14-8-32_s004.docx]

**Supplementary Table 3. Association of MPOD patterns with AREDS stages stratified by gender**

| **Characteristic** |  | |  | |  |  | ***Obana pattern*** | | | | | |  | ***P-value*** |
| --- | --- | --- | --- | --- | --- | --- | --- | --- | --- | --- | --- | --- | --- | --- |
|  | | *total* | | *Peak* | |  | | *Ring* |  | *Mixed* |  | *Dip* |  |  |
| AREDS grade, n (%) | |  | |  | |  | |  |  |  |  |  |  |  |
| Normal | | 427 | | 289 | | 67.7% | | 81 | 19.0% | 23 | 5.4% | 34 | 8.0% |  |
| Early AMD | | 224 | | 156 | | 69.6% | | 37 | 16.5% | 18 | 8.0% | 13 | 5.8% | 0.45 |
| AREDS grades, males, n (%) | |  | |  | |  | |  |  |  |  |  |  |  |
| Normal | | 146 | | 105 | | 71.9% | | 19 | 13.0% | 9 | 6.2% | 13 | 8.9% |  |
| Early AMD | | 97 | | 75 | | 77.3% | | 9 | 9.3% | 9 | 9.3% | 4 | 4.1% | 0.39 |
| AREDS grade, females, n (%) | |  | |  | |  | |  |  |  |  |  |  |  |
| Normal | | 281 | | 184 | | 65.5% | | 62 | 22.1% | 14 | 5.0% | 21 | 7.5% |  |
| Early AMD | | 127 | | 81 | | 63.8% | | 28 | 22.1% | 9 | 7.1% | 9 | 7.1% | 0.86 |
