# Supplementary material for: Advanced Analysis Tools for Two Wavelength Autofluorescence Imaging of Macular Xanthophyll Carotenoids: ALSTAR2 Baseline
Source: Transl Vis Sci Technol. 2025 Aug 21;14(8):32. doi: 10.1167/tvst.14.8.32 (PMC12393178; doi:10.1167/tvst.14.8.32)

Supplementary Figure 1. Comparison of grading by author LG for centering the MPOD distribution

Axes indicate distance from the manually determined center in µm, applying 11.3 µm per pixel. The plot shows that most points lie within the 95% limits (red dashed circle) of agreement, indicating acceptable agreement. A few outliers suggest occasional disagreement, but not systematic bias.

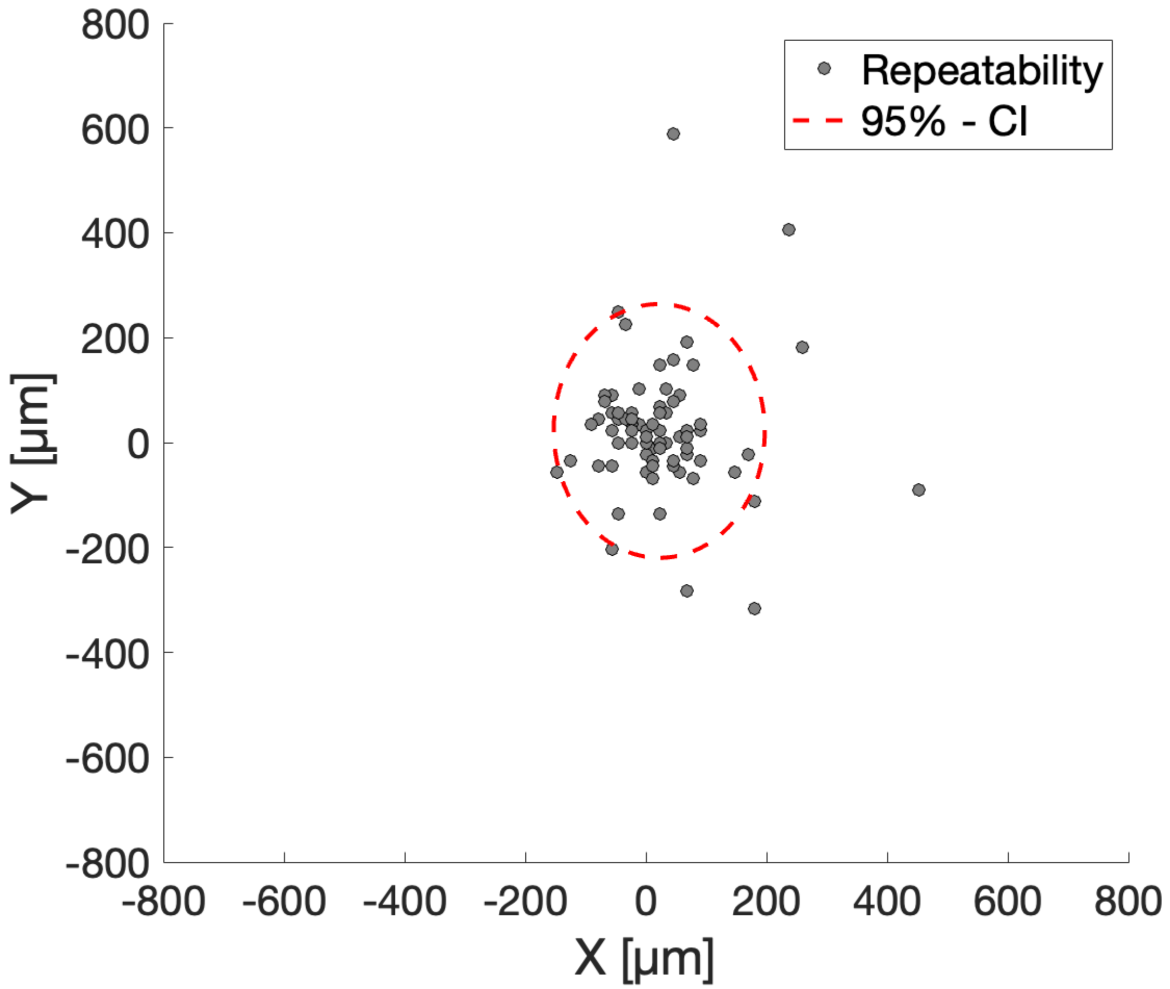

Supplement: Supplement 5 [file tvst-14-8-32_s005.pdf]
